# Palonosetron, a 5-HT3 Receptor Antagonist, Induces G1 Cell Cycle Arrest and Autophagy in Gastric Cancer Cells

**DOI:** 10.3390/ijms262010039

**Published:** 2025-10-15

**Authors:** Young Chul Yoo, Lin Lin, Sihak Lee, Yeeun Rachel Shin, Ju Eun Oh, Na Young Kim

**Affiliations:** 1 Department of Anesthesiology and Pain Medicine, Anesthesia and Pain Research Institute, Yonsei University College of Medicine, 50-1 Yonsei-ro, Seodaemun-gu, Seoul 03722, Republic of Korea; seaoyster@yuhs.ac (Y.C.Y.); linlin@yuhs.ac (L.L.); 2 Stanley Center for Psychiatric Research, Broad Institute of Massachusetts Institute of Technology and Harvard, Cambridge, MA 02142, USA; leesihak@broadinstitute.org; 3 Sha Tin College, No. 3 Lai Wo Lane, Fo Tan, Sha Tin, New Territories, Hong Kong; shiny3@stconline.edu.hk

**Keywords:** 5-HT3 receptor, palonosetron, gastric cancer, G1 cell cycle

## Abstract

Serotonin or 5-hydroxytryptamine (5-HT) has been implicated in promoting cancer cell growth by acting on 5-HT receptors, such as 5-HT1 and 5-HT2 receptors. However, the role of 5-HT3 receptor antagonists in gastric cancer cell lines remains unclear. This study aimed to evaluate the effect of 5-HT3 receptor antagonists (ondansetron, palonosetron, and ramosetron) on cancer cell growth using AGS and MKN-1 cell lines, as well as the xenograft mouse model. All the three antagonists inhibited cell proliferation, migration, and colony formation in AGS cells. Specifically, palonosetron induced G1 cell cycle arrest, autophagy, and phosphorylation of GSK3β, along with increased expression of p27, p53, and LC3B. In vivo studies demonstrated that palonosetron reduced tumor growth and modulated pro-inflammatory cytokines—tumor necrosis factor alpha, interleukin 6, and interleukin 1β. These findings suggest that 5-HT3 receptor antagonists, especially palonosetron, exert anti-tumor effects in gastric cancer through G1 cell cycle regulation and immunomodulation. The results position palonosetron as a promising lead for further preclinical development in gastric cancer.

## 1. Introduction

Serotonin, also known as 5-hydroxytryptamine (5-HT), is a monoamine neurotransmitter synthesized from tryptophan [1]. In humans and animals, it is abundant in the gastrointestinal tract, platelets, and the central nervous system [2,3]. In particular, approximately 90% of the body’s total serotonin is stored in enterochromaffin cells within the gastrointestinal tract and plays an important role in regulating gastrointestinal (GI) motility [4,5]. Serotonin is widely recognized for the regulation of mood and promotion of well-being and happiness [2,3]. Moreover, it is associated with appetite, sleep, learning, and memory [6]. The regulation of serotonin synthesis in serotonergic neurons of the central nervous system underlies its pharmacological classification as an antidepressant [7]. Serotonin also acts as a growth factor for some cells, inducing vasoconstriction, and is involved in homeostasis, blood coagulation, and wound healing [8,9,10].

In addition, serotonin has been reported to act as a mitogenic factor in both normal and cancer cells [11]. It also promotes tumor growth in various cancers, including prostate, lung, and colorectal cancers, by acting on 5-HT receptors. In particular, 5-HT1 and 5-HT2 receptors are closely associated with aggressive tendencies of cancer cells [12,13], prompting extensive research on the inhibitory potential of 5-HT1 and 5-HT2 receptor antagonists against cancer cell growth [14,15]. To date, seven 5-HT receptors have been identified, of which only 5-HT3 receptor is not a G-protein-coupled receptor and it activates extrinsic sensory nerves via the sodium-potassium pump [16].

In clinic, 5-HT3 receptor antagonists are currently the most widely used drugs against postoperative nausea and vomiting (PONV) [17], and they include azasetron (Serotone, Welfide, Japan), dolasetron (Anzemet, Sanofi-Aventis, France), granisetron (Kytril, Roche, Switzerland), tropisetron (Navoban, Novartis, Switzerland), and ondansetron (Zofran, GlaxoSmith-Kline, England) [18]. Studies on 5-HT receptor antagonists have focused on their anticancer effects in various cancer types and an efficacy comparison of related drugs for PONV prevention [19,20]. However, the role of 5-HT3 receptor and its antagonism in cancer development has not been widely explored. Therefore, this study aimed to identify the dose-dependent effects of a 5-HT3 receptor antagonists on gastric cancer cells. The in vitro experiment used gastric cancer cell lines—AGS and MKN-1—and the in vivo experiment employed a xenograft mouse model.

Glycogen synthase kinase 3 (GSK3) shows various function in cellular process including differentiation, growth, motility and apoptosis [21]. Multiple studies have been developed to modulate GSK3β activity in order to suppress cancer growth [22,23,24,25]. GSK3β mediates signaling of p27, p53, and LC3B. The CDK inhibitor p27 and the transcription factor p53 suppress tumor by arresting cell-cycle progression and inducing apoptosis. Upregulating p27 enforces the G1/S checkpoint and often coincides with apoptosis in cancer cells, whereas activated p53 triggers cell-cycle arrest and apoptosis [26,27,28,29,30]. LC3B is a core autophagosome component. The conversion from LC3-I to LC3-II tracks autophagosome formation [31,32]. In summary, modulation of GSK3β impacts p27 and p53-mediated cell-cycle arrest and apoptosis, while coupling to LC3B-dependent autophagy.

**Scheme 1:**
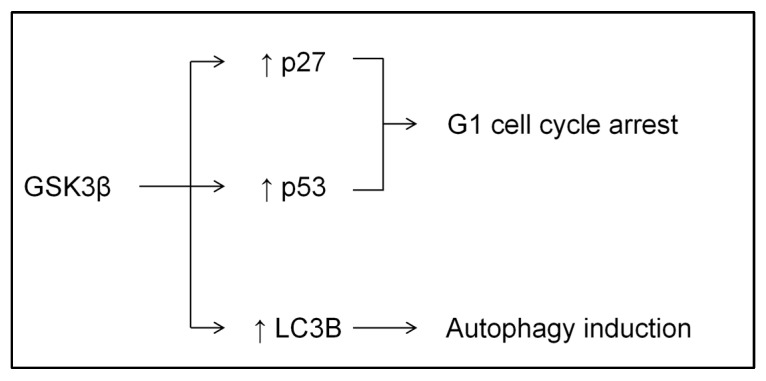
Schematic representation of signaling interactions among GSK3, cell-cycle progression, and autophagy. Arrows indicate the direction of effect and the resulting outcome.

Scheme 1 provides a schematic overview of these interrelationships.

## 2. Results

### 2.1. 5-HT3 Receptor Antagonists Inhibit Cell Proliferation in Human Gastric Cancer Cells

**Figure 1:**
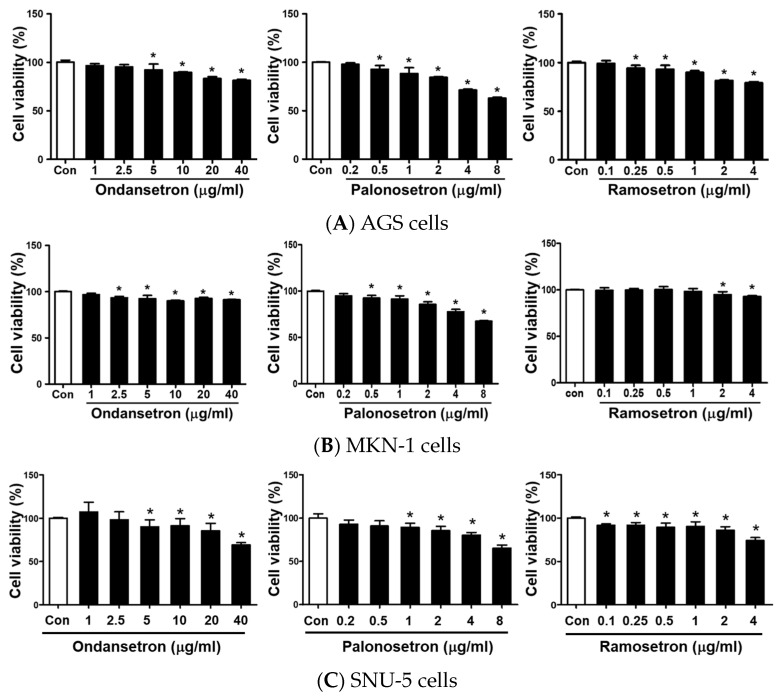
5-Hydroxytryptamine 3 (5-HT3) receptor antagonists inhibited proliferation of AGS, MKN-1, and SNU-5 cells. 5-HT3 receptor antagonists reduced the viability of AGS (**A**) and MKN-1 cells (**B**), as measured using EZ-Cytox cell viability assay kit. Statistical significance was assessed using one-way analysis of variance with the Bonferroni correction. *n* = 6, * *p* < 0.05 compared with the saline-treated control group. AGS, MKN-1, and SNU-1, Human gastric adenocarcinoma cell lines.

To determine the effects of 5-HT3 receptor antagonists on the viability of human gastric cancer cells, AGS, MKN-1, and SNU-5 cells were initially treated with different concentrations of ondansetron (0–40 μg/mL), palonosetron (0–8 μg/mL), and ramosetron (0–4 μg/mL) in complete cell culture media for 48 h prior to the cell proliferation assay. Compared with the saline-control, cell viability (%) at day 2 reduced in a dose-dependent manner after the three 5-HT3 receptor antagonist treatments (Figure 1).

### 2.2. 5-HT3 Receptor Antagonists Prevent Cell Migration and Colony Formation in AGS

**Figure 2:**
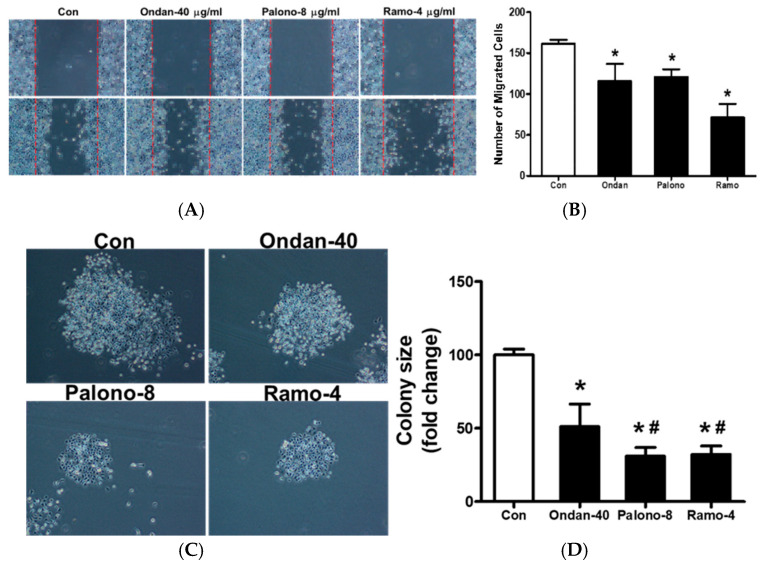
5-Hydroxytryptamine 3 (5-HT3) receptor antagonists inhibited cell migration and colony formation in AGS cells. (**A**) The representative images of wound healing scratch assay. The dashed red line indicates the location of the initial scratch. (**B**) The quantitation of the migrated cells demonstrated that 5-HT3 receptor antagonists suppressed AGS cell migration. *n* = 5, * *p* < 0.05 compared with the saline-treated control group. (**C**) The representative images of colony formation. (**D**) Colony size was measured using the ImageJ, version 1.44 software program; * *p* < 0.05 vs. control, # *p* < 0.05 vs. ondansetron 40 g/mL. Con, control; Palono-8, Palonosetron 8 μg/mL; Ondan-40, Ondansetron 40 μg/mL; Ramo-4, Ramosetron 4 μg/mL.

The effects of 5-HT3 receptor antagonists on AGS cell migration were investigated via wound healing and colony formation assays. The saline-control group showed a reduction in the width of the wound after 12 or 36 h as cells migrated into the wound. Compared with the saline-control, the wound width was slightly reduced in cells treated with the three 5-HT3 receptor antagonists. Ondansetron, palonosetron, and ramosetron reduced migrated cells 72 ± 13%, 75 ± 6%, and 44 ± 10%, respectively, compared with the control (100%) (*p* < 0.05) (Figure 2A,B). An increase in migration was only observed in the control group after 12 h. (Supplementary Figure S1).

In the colony formation assay, the saline-control group showed colony formation increase after 9 days. Compared with the saline-control (100%), ondansetron, palonosetron and ramosetron decreased colony formation by 51 ± 15%, 31 ± 5.8%, and 32 ± 5.9%, respectively (*p* < 0.05) (Figure 2C,D).

### 2.3. Palonosetron Arrested G1 Phase in AGS

**Figure 3:**
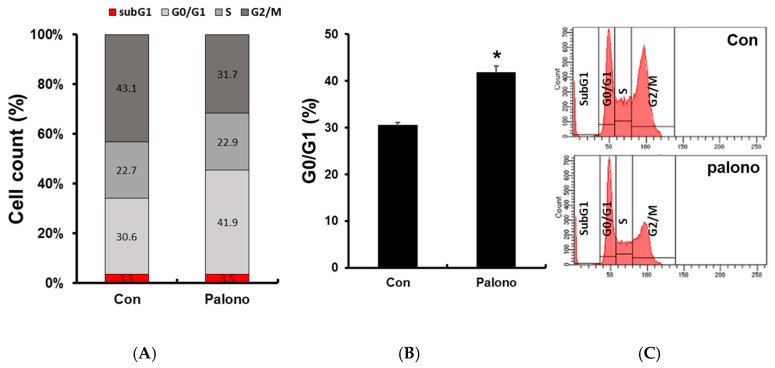
Palonosetron induced G1 phase arrest in AGS cells. Flow cytometry analysis showed that palonosetron treatment increased AGS cell cycle arrest, as determined by the quantitation of cells in each cell cycle phase. (**A**) Quantitation of cells in the G1 phase; y-axis shows percentage of cells in each phase. (**B**) Cell cycle assessment; y-axis shows percentage of cells in the G1 phase. (**C**) X-axis shows propidium iodide, whereas y-axis shows the count of cell cycle. Statistical significance was assessed using one-way analysis of variance with Bonferroni correction. * *p* < 0.05 compared with the saline-treated control group. Con, control; Palono, Palonosetron.

We conducted a cell cycle assay via flow cytometry to investigate the inhibitory activities of Palonosetron on the gastric cancer cell proliferation. Palonosetron treatment (8 µg/mL) for 24 h significantly increased the percentage of cells in the G1 phase to 41.9 ± 1.3% compared with 30.6 ± 0.5% % in the control (*p* < 0.05) (Figure 3).

### 2.4. 5-HT3 Receptor Antagonists Stimulated Phosphorylation of GSK3, ERK, and AKT and Autophagy-Related Proteins in AGS

**Figure 4:**
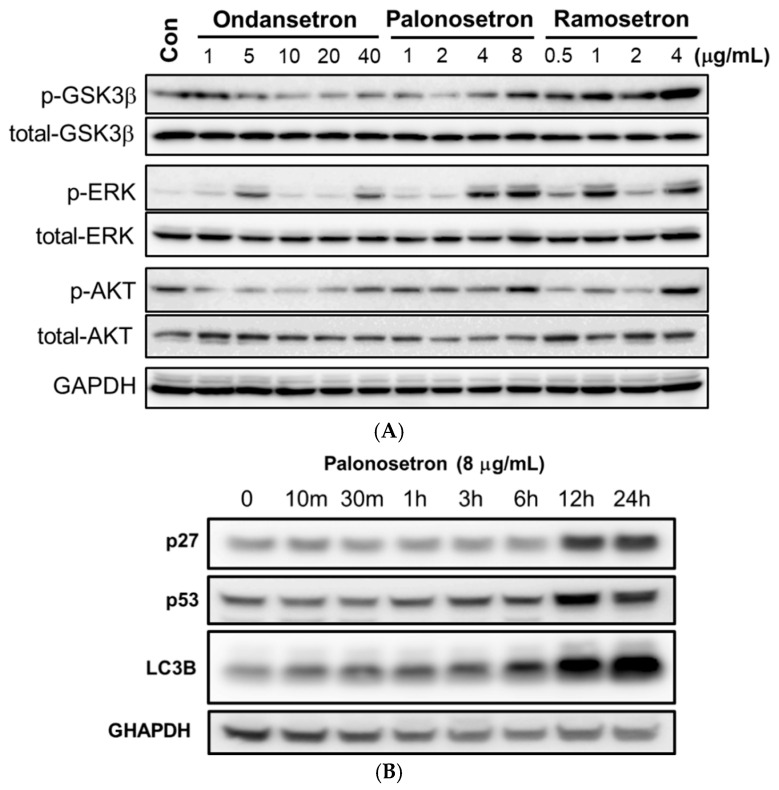
5-Hydroxytryptamine 3 (5-HT3) receptor antagonists stimulated GSK3β, ERK, and AKT phosphorylation and upregulated p27, p53, and LC3B expression (**A**) Western blot analysis showed that 5-HT3 receptor antagonists increased GSK3β, ERK, and AKT phosphorylation. (**B**) Western blot analysis showed that palonosetron treatment increased the protein levels of p27, p53, and LC3B in AGS cells relative to the saline-treated control. GAPDH was used for normalizing protein expression. GSK3β, glycogen synthesis kinase 3 beta; ERK, extracellular signal-regulated kinase; AKT, protein kinase B; LC3B, microtubule-associated protein 1A/1B-light chain 3; GAPDH, glyceraldehyde-3-phosphate dehydrogenase.

To explore the potential cellular signaling pathway by which 5-HT3 receptor antagonists affected AGS cell viability and migration, we investigated the involvement of the GSK3 pathway. Ondansetron, palonosetron, and ramosetron increased the phosphorylation of GSK3, ERK, and AKT in AGS (Figure 4A).

Furthermore, autophagy has been shown to play a critical role in the fate of cancer cells, including initiation, progression, and apoptosis [33,34]. We examined whether 5-HT3 receptor antagonists affected autophagy in AGS cells. Compared with the saline-control, palonosetron treatment enhanced the levels of p27, p53, and LC3B in a time-dependent manner in AGS (Figure 4B).

### 2.5. Palonosetron Regulated Rb, p27, p53, and LC3B via the Phosphorylation of GSK3β in AGS

**Figure 5:**
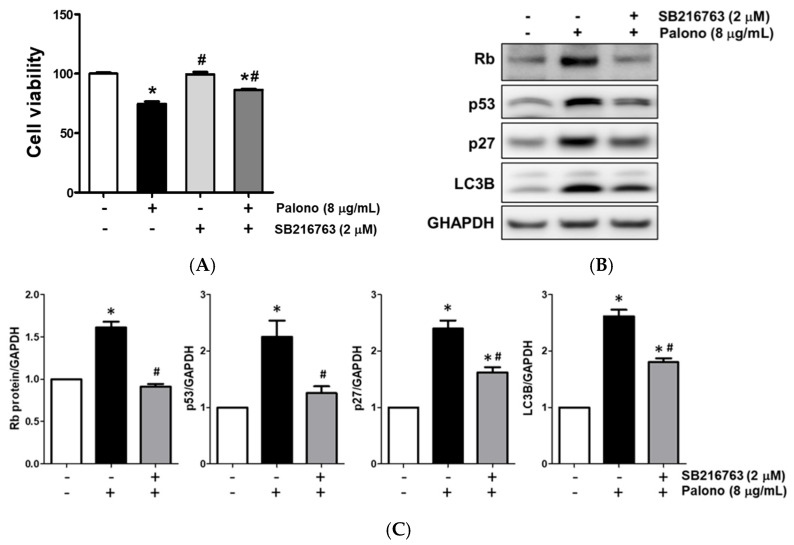
Palonosetron regulated the expression of Rb, p27, p53, and LC3B via GSK3β phosphorylation in AGS cells. (**A**) Cell viability analysis showed that the co-treatment of cells with palonosetron (8 μg/mL) and SB216763 (2 μM) abolished the effect of palonosetron on AGS cell viability. (**B**) Western blot analysis demonstrated that palonosetron treatment increased the protein expression of Rb, p53, p27, and LC3B in AGS cells, whereas co-treatment with palonosetron (8 μg/mL) and SB216763 (2 μM) decreased their expression. (**C**) Expression of each protein was normalized using GAPDH. Data are expressed as relative values, with untreated cells as 1. * *p* < 0.05 compared with the saline-treated control group. # *p* < 0.05 compared with the palonosetron-treated group. Rb, retinoblastoma; SB216763, GSK3β inhibitor; Palono, palonosetron; GAPDH, glyceraldehyde-3-phosphate dehydrogenase. - means no drug treatment, + means drug treatment.

To confirm whether Palonosetron affected AGS cell viability via the GSK3β signaling pathway, we blocked the pathway using SB216763, a specific inhibitor of GSK3β. Compared with the control, SB216763 treatment did not change AGS cell viability, whereas palonosetron (8 µg/mL) treatment decreased AGS cell viability. Co-treatment with SB216763 and palonosetron attenuated palonosetron-induced AGS cell viability reduction (Figure 5A). Co-treatment with SB216763 and palonosetron decreased the protein levels of Rb, p27, p53, and LC3B compared with palonosetron treatment only (Figure 5B). The results of our experiment shown in Figure 5B are presented as a histogram in Figure 5C.

### 2.6. Palonosetron Induced Autophagy

Bafilomycin A1 (Baf-A1) increased LC3 expression by inhibiting lysosomal degradation, whereas 3-methyladenine (3-MA) decreased LC3 levels by suppressing autophagosome formation.

To further investigate autophagy induced by palonosetron, AGS cells were treated with palonosetron for 24 h. Then, 50 nM Baf-A1 was added 4 h before harvest. Western blot analysis was subsequently performed to assess protein expression.

**Figure 6:**
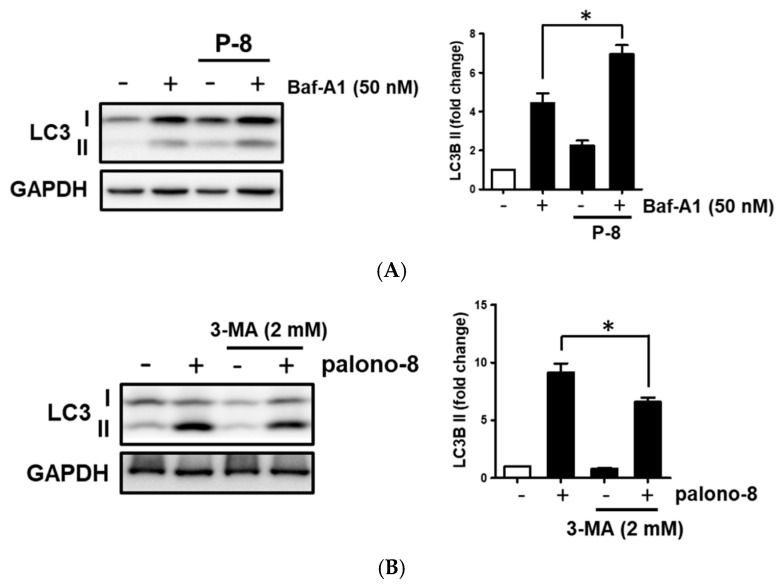
Palonosetron induced autophagy in AGS cells. (**A**) Western blot analysis showed that co-treatment of AGS cells with Palonosetron and Baf-A1 (50 nM) increased LC3B expression to a greater extent than that with Palonosetron alone. (**B**) Western blot analysis showed that treatment of AGS cells with Palonosetron alone increased the protein expression of LC3B, whereas co-treatment with Palonosetron and 3-MA (2 mM) decreased its expression. GAPDH served as a loading control. Baf-A1, bafilomycin A1; 3-MA, 3-methyladenine; GAPDH, glyceraldehyde-3-phosphate dehydrogenase; Palono-8, Palonosetron 8 μg/mL. * *p* < 0.05 compared with the saline-treated control group. - means no drug treatment, + means drug treatment.

The results revealed that Baf-A1 stimulated the expression of autophagy-related protein (LC3B) in AGS (Figure 6). Moreover, co-treatment with Baf-A1 and palonosetron markedly enhanced the protein level of LC3B compared with Baf-A1 treatment alone. The results for ramosetron and ondansetron were similar to those of palosetron (Supplementary Figure S5).

### 2.7. Palonosetron Reduced the Tumorigenesis of AGS Cells in the Gastric Cancer Xenograft Models

**Figure 7:**
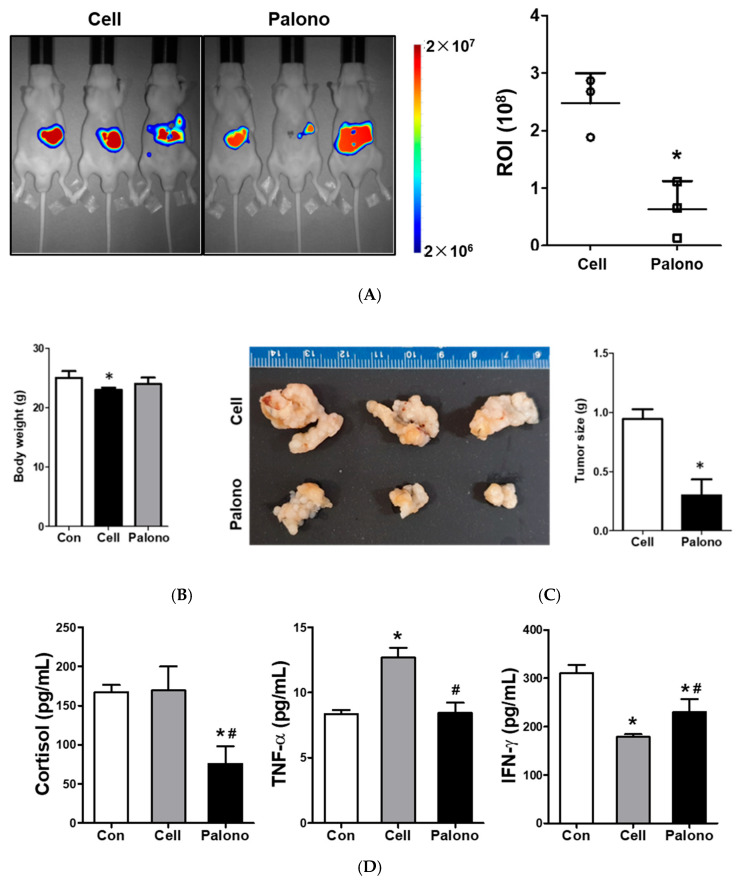
(**A**) Palonosetron decreased tumor size in nude mice injected with AGS-Luc cells. In vivo imaging and corresponding individual data points in the bar graph show the ROI measurements for the two groups, reflecting tumor burden. Values are presented as means and SD. *n* = 8–10. These experiments were performed in triplicate. * *p* < 0.05 vs. cell group. (**B**) The body weight of each experimental group was measured. (**C**) After the experiment, the weight of the tumor masses was measured. (**D**) Effects of palonosetron on circulating levels of inflammatory cytokines. Blood serum levels of cortisol, TNF-α, and IFN-γ were measured using an ELISA kit. *n* = 7–10 per group. Values are presented as means and standard deviation. * *p* < 0.05 vs. con group, # *p* < 0.05 vs. cell group. ROI, region of interest; Con, normal saline group; Cell, AGS-luc cells-injected group; Palono, palonosetron-treated AGS-luc-injected group. TNF-α, tumor necrosis factor α; IFN-γ, interferon γ; ELISA, enzyme-linked immunosorbent assay.

We used xenograft models to investigate the effects of 5-HT3 receptor antagonist (palonosetron) on the tumor growth of AGS-luc cells. Four weeks after subcutaneous injection of AGS-luc cells, IVIS imaging revealed a significant reduction in tumor volume in the palonosetron-treated group compared with the saline-treated controls (*p* < 0.05). Fluorescence intensity, ranging from blue (low) to red (high), corresponded to tumor burden, which was quantified using the ROI analysis (Figure 7A). The body weight of nude mice significantly reduced in the group injected with only AGS-Luc cells (Figure 7B). The tumor weight was significantly reduced in mice treated with PALLA compared with that in mice injected with only AGS-Luc cells (Figure 7C).

Compared with the saline-control, palonosetron significantly decreased the serum level of cortisol by 45 ± 14% of control (*p* < 0.05). The serum level of TNF-α was also significantly decreased by palonosetron (64.6 ± 6% of the saline-control, *p* < 0.05). Further, the serum level of IFN-γ was significantly increased by palonosetron (128 ± 14.5% of the saline-control, *p* < 0.05) (Figure 7D).

Throughout the treatment, mice maintained stable body weight and no deaths occurred.

## 3. Discussion

Palonosetron, commonly known for its efficacy in combating chemotherapy-induced nausea, has emerged as a potential agent in modifying tumor pathology, particularly in reducing tumor size and weight [35,36]. This intriguing development is further accentuated by its impact on key inflammatory markers. Studies have reported a consistent decrease in the serum levels of pro-inflammatory cytokines, such as TNF-α, IL-6, and IL-1β, following palonosetron administration, suggesting an immunomodulatory effect that might contribute to its anti-tumoral properties [14]. The reduced level of these cytokines, which are instrumental in promoting a pro-tumorigenic environment, hints at a broader therapeutic scope of palonosetron, possibly extending its role from symptomatic relief to a more active participation in tumor suppression [15]. These observations necessitate a deeper exploration of the mechanistic pathways of palonosetron that underlie its dual-function profile, which synergizes its antiemetic efficacy with a direct anti-cancer effect. Future research should focus on elucidating the exact molecular interactions of palonosetron in the tumor microenvironment and determining its clinical relevance in comprehensive cancer management strategies.

The cell viability assay results showed that palonosetron reduces the viability of AGS cells in a dose-dependent manner within 48 h, suggesting its potential cytotoxic effects against gastric cancer cells. This observed decrease in cell proliferation could be indicative of an anti-cancer activity that warrants further exploration, particularly because AGS cells are derived from a gastric adenocarcinoma and are commonly used to study gastric cancer therapies. The mechanism underlying this palonosetron effect on cell viability is likely related to its role as a serotonin receptor antagonist [16]. Serotonin is known to influence cell proliferation, and thus its inhibition may disrupt cancer cell growth [17]. Of note, this suppression effect on cell proliferation is dose-dependent, highlighting the potential of tailoring palonosetron’s concentration to maximize therapeutic efficacy while minimizing toxicity. Given these findings, the broader implications of using palonosetron as an antineoplastic agent necessitate further exploration. Subsequent research should delineate the exact pathways through which palonosetron influences AGS cell viability, evaluate its efficacy across a spectrum of cancer cell lines, and establish its in vivo antitumor potential. These efforts will clarify whether palonosetron can be repurposed as a viable option in gastric cancer treatment protocols, alongside or as an alternative to existing chemotherapy agents.

In addition, the results showed that palonosetron arrests the cell cycle of AGS cells in the G1 phase, a critical stage for cell growth and preparation for DNA replication, which was associated with upregulation of cell cycle inhibitors p27 and p53, and modulation of Rb phosphorylation [37]. In the G1 phase, cells decide whether to enter the cycle and proceed with division or enter a quiescent state (G0) [38]. The ability of palonosetron to halt cell growth in this phase suggests its potential to impede cell cycle progression, thereby inhibiting cell proliferation—a desirable effect in cancer treatment. This cell cycle arrest could be an effect of palonosetron on signaling pathways that regulate cell cycle progression. For instance, palonosetron may affect cyclin-dependent kinases and cyclins, which are crucial for the transition from G1 to S phase [39]. By halting cells in the G1 phase, palonosetron might induce a state of dormancy in cancer cells, reducing the rate of tumor growth. Furthermore, it may be indicative of its potential to induce cellular senescence or promote differentiation, both of which are therapeutic strategies in cancer treatment [40,41]. Given that AGS cells are a model for gastric cancer, these findings could directly influence the development of new treatments for this type of cancer. However, further research is warranted to determine whether the G1 arrest is reversible, or it leads to apoptosis upon prolonged exposure to palonosetron. Subsequent studies should elucidate the molecular mechanisms by which palonosetron induces cell cycle arrest, as well as evaluate its efficacy in vivo. Such research could provide a scientific foundation for clinical trials assessing the effectiveness of palonosetron as a stand-alone treatment or in combination with other therapeutic agents.

Across assays, all three antagonists (ondansetron, palonosetron, and ramosetron) suppressed proliferation and migration to some degree. However, only palonosetron consistently triggered G1-phase arrest and autophagy features, consistent with its distinct receptor interaction. In contrast, ondansetron and ramosetron remained cytostatic consistent G1 arrest or autophagy at equal exposures, implicating drug-specific signaling bias downstream of 5-HT3R and motivating targeted mechanistic studies and optimization.

We observed that palonosetron enhanced phosphorylation of GSK3β, ERK and AKT, suggesting activation of intracellular signaling cascades that intersect with both cell cycle and autophagy regulation [42]. The treatment of AGS cells with Palonosetron alone increased the protein expression of LC3B, suggests that palonosetron promotes autophagic responses. Using SB216763 reversed the upregulation of p27, p53, and LC3B, implicating the phosphorylation of GSK3β as a key mediator for palonosetron’s effect.

The in vivo xenograft experiments support the in vitro findings, demonstrating the palonosetron administration significantly reduced cancer size in AGS-luc cells-injected mice. The palonosetron administration also induced the reduction in circulating cortisol and TNF-α levels, along with the increase in IFN-γ levels, indicating that palonosetron not only directly inhibits tumor growth but also modulates systemic inflammatory and immune responses. Compared with the control group, palonosetron-treated mice exhibited no weight loss and treatment-related mortality, indicating no overt toxicity at the tested dose.

**Scheme 2:**
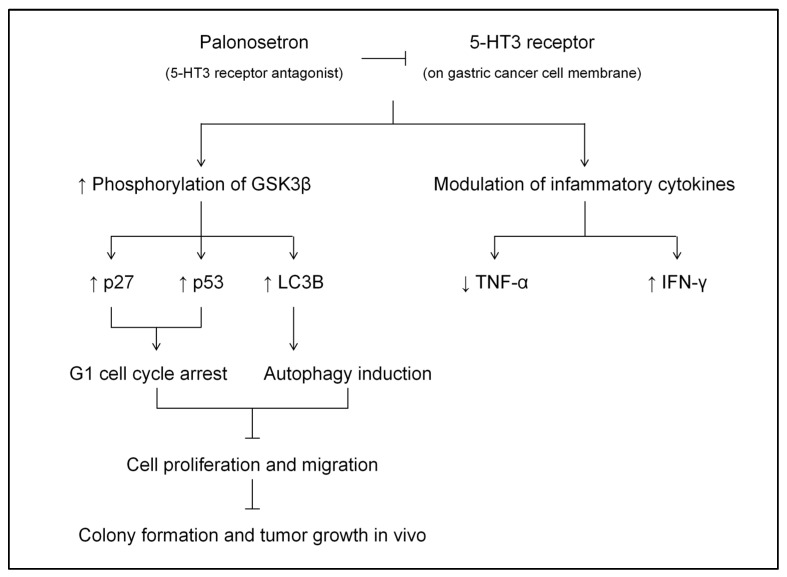
Proposed mechanism of Palonosetron-mediated inhibition of gastric cancer progression. Arrows denote the direction of effect and the resulting outcome. Blunt-ended lines (T-bars) denote inhibition.

The integrated mechanism is illustrated in Scheme 2.

Our data show that palonosetron induces G1-phase arrest and engages autophagy in gastric cancer cells, with concordant attenuation of tumor cell proliferation, migration and tumor growth. Compared with studies of other 5-HT3 antagonists that primarily document anti-proliferative effects without robust G1 arrest [43,44,45], our findings suggest a drug-specific bias for palonosetron toward cell-cycle and autophagy modulation.

These findings suggest palonosetron as a promising lead for further investigation into gastric cancer. Additional mechanistic, pharmacology, and safety studies are required prior to therapeutic positioning. The antitumor effect is caused by inhibition of cell proliferation through G1 arrest and GSK3β-dependent autophagy induction, and modulation of inflammatory cytokines that may enhance antitumor immunity. Further studies should investigate its efficacy in combination with standard chemotherapy or other agents of gastric cancer.

## 4. Materials and Methods

### 4.1. Cell Culture

SNU-5, AGS and MKN-1 cell lines were chosen. SNU-5 cell line exhibits suspension growth. AGS and MKN-1 are two widely used adherent lines. for proliferation assays. AGS was used for signaling studies due to its consistent performance in pathway readouts. SNU-5, AGS and MKN-1, purchased from the American Type Culture Collection (Rockville, MD, USA), which are human gastric adenosquamous carcinoma cell lines, were maintained in Rosewell Park Memorial Institute (RPMI) 1640 medium supplemented with 10% fetal bovine serum (FBS), 100 unit/mL penicillin, and 100 µg/mL streptomycin at 37 °C in 95% humidified air plus 5% CO_2_. Culture media and all supplements were purchased from Gibco (Middletown, VA, USA). Ondansetron, palonosetron, and ramosetron were purchased from Cell Signaling Technology (Danvers, MA, USA) and Sigma-Aldrich (St. Louis, MO, USA).

### 4.2. Cell Viability Assay

SNU-5, AGS and MKN-1 (3X103) cells were seeded into 96-well culture plates and incubated at 37 °C overnight. The cells were then incubated at 37 °C for 2 days in RPMI supplemented with 10% FBS and ondansetron, palonosetron, and ramosetron, respectively, at indicated concentrations. Cell viability test was performed using the EZ-Cytox-enhanced cell viability assay kit (DoGenBio, Seoul, Republic of Korea), according to the manufacturer’s protocol. These experiments were performed in triplicate.

### 4.3. Wound Healing Assay

For the wound healing assay, a linear scratch wound was created in monolayers of AGS cells at 80–90% confluence using a 200 µL plastic pipette tip, followed by the separate addition of ondansetron (40 μg/mL), palonosetron (8 μg/mL), and ramosetron (4 μg/mL) or none. After 24 h incubation at 37 °C, the wound was analyzed under a microscope, images were captured, and migrated cells were counted. These experiments were performed in triplicate.

### 4.4. Colony Formation Assay

The effect of three 5-HT3 receptor antagonists on the proliferation of AGS cells was analyzed using a clonogenic assay. Briefly, AGS cells were seeded on 6-well plates at a density of 300 cells/well. Cells were incubated at 37 °C in the presence or absence of ondansetron (40 μg/mL), palonosetron (8 μg/mL), and ramosetron (4 μg/mL). After 10 days of culture, colonies were fixed in 2% paraformaldehyde for 10 min and photographed, and then colony sizes were measured. These experiments were performed in triplicate.

### 4.5. Cell Cycle Assay

Cell cycle was examined via flow cytometry by staining cells with propidium iodide (PI), as previously described [46]. Briefly, following treatment with palonosetron, AGS cells were trypsinized, washed with phosphate-buffered saline (PBS), fixed in cold 70% ethanol for 30 min at 4 °C, and then washed twice with PBS. Fixed cells were incubated at room temperature for 20 min with 50 μg/mL RNase A, 50 μg/mL PI was added, and the solution was incubated for another 20 min. The labeled cells were measured immediately using a BD FACScan flow cytometer (BD Biosciences, San Jose, CA, USA). The acquired data were analyzed with CellQuest Pro version 5.1 software (BD Biosciences). These experiments were performed in triplicate.

### 4.6. Western Blot Analysis

To study the dose and time-response effects of ondansetron, palonosetron, and ramosetron on the cell cycle checkpoint proteins and autophagy, cells were treated with three 5HT-3 receptor antagonists or none, cultured for 24 h, and washed twice with ice-cold PBS. Total protein extraction and immunoblotting were performed, as previously described [33]. The following primary antibodies were used: anti-phospho-GSK3β (serine 9), anti-GSK3β, anti-phospho-ERK (threonine 202/tyrosine 204), anti-ERK, anti-phospho-AKT (serine 473), anti-AKT, anti-Rb, anti-p27, anti-p53, anti-LC3B (all from Cell Signaling Technology, Danvers, MA, USA), and anti-glyceraldehyde-3-phosphate dehydrogenase (GAPDH) (Invitrogen, Carlsbad, CA, USA). These experiments were performed in triplicate. The original, uncropped Western blot membrane is presented in Supplementary Figures S2–S5.

### 4.7. Xenograft Models

All animal procedures were approved by the committee for the Care and Use of Laboratory Animals of Yonsei University College of Medicine (IACUC 2017-0226) and were conducted according to the guidelines of the Care and Use of Laboratory Animals (US National Institutes of Health). Six-week-old male BALB/c nude mice (weighing 17–18 g) were used in these experiments. For the mouse tumor xenograft model, 20 BALB/c nude mice were injected subcutaneously with AGS-Luc cells (5 × 10^6^ cells in 0.1 mL PBS). They were then randomly divided into either saline (*n* = 10) or palonosetron (100 μg/kg/day) treatment groups (*n* = 10) to evaluate the response of the metastatic gastric tumor xenografts to palonosetron. The drug-loaded pump was implanted subcutaneously into the left flank of BALB/c nude mice under isoflurane anesthesia. No procedural mortality occurred. Treatment was delivered using a micro-osmotic pump system (ALZET model 1004, DURECT Corporation, Cupertino, CA, USA) at a flow rate of 0.11 μL/h for 28 days. Treatment and tumor cell implantation occurred concurrently. After four weeks, imaging was performed using an IVIS imaging system, and nude mice were sacrificed. All fluorescence images were acquired with a 15 min exposure. For quantitative comparison, regions of interest (ROIs) were drawn over the tumor, and the results are expressed as means ± standard deviations (*n* = 8). These experiments were performed in triplicate.

### 4.8. Enzyme-Linked Immunosorbent Assay

Enzyme-linked immunosorbent assay commercial kits (R&D Systems, Minneapolis, MN, USA) were used to measure serum levels of cortisol, interferon (IFN)-γ and tumor necrosis factor (TNF)-α in mouse xenograft models, according to the manufacturer’s instructions. These experiments were performed in triplicate.

### 4.9. Statistical Analysis

The results of the in vitro and in vivo assays are presented as means ± standard deviations. Statistical significance was determined using one-way analysis of variance or Student’s *t*-test following the Bonferroni correction. Statistical analyses were conducted using Prism 5.01 software (GraphPad, San Diego, CA, USA). A *p*-value < 0.05 was considered statistically significant.

## 5. Conclusions

Palonosetron, a 5-HT3 receptor antagonist, inhibited gastric cancer progression by suppressing cell proliferation, migration, and colony formation via induction of G1 cell cycle arrest and promoting autophagy through activation of the GSK3β. In vivo, palonosetron not only significantly reduced tumor growth but also modulated inflammatory cytokines.

These findings identify palonosetron as a lead compound for further investigation in gastric cancer. Future studies are warranted to further explore the underlying molecular mechanisms and evaluate the clinically applicability of palonosetron in gastric cancer.

## Data Availability

The datasets used or analyzed in the current study are available from the corresponding authors on reasonable request.

## Supplementary Materials

The following supporting information can be downloaded at: https://www.mdpi.com/article/10.3390/ijms262010039/s1.

## Abbreviations

The following abbreviations are used in this manuscript:

5-HT	5-hydroxytryptamine
Baf-A1	Bafilomycin A1
FBS	fetal bovine serum
GAPDH	glyceraldehyde-3-phosphate dehydrogenase
GI	gastrointestinal
IFN	interferon
PI	propidium iodide
PONV	postoperative nausea and vomiting
ROIs	regions of interest
TNF	tumor necrosis factor
