# Supplementary figures and images for: Palonosetron, a 5-HT3 Receptor Antagonist, Induces G1 Cell Cycle Arrest and Autophagy in Gastric Cancer Cells
Source: Int J Mol Sci. 2025 Oct 15;26(20):10039. doi: 10.3390/ijms262010039 (PMC12562317; doi:10.3390/ijms262010039)

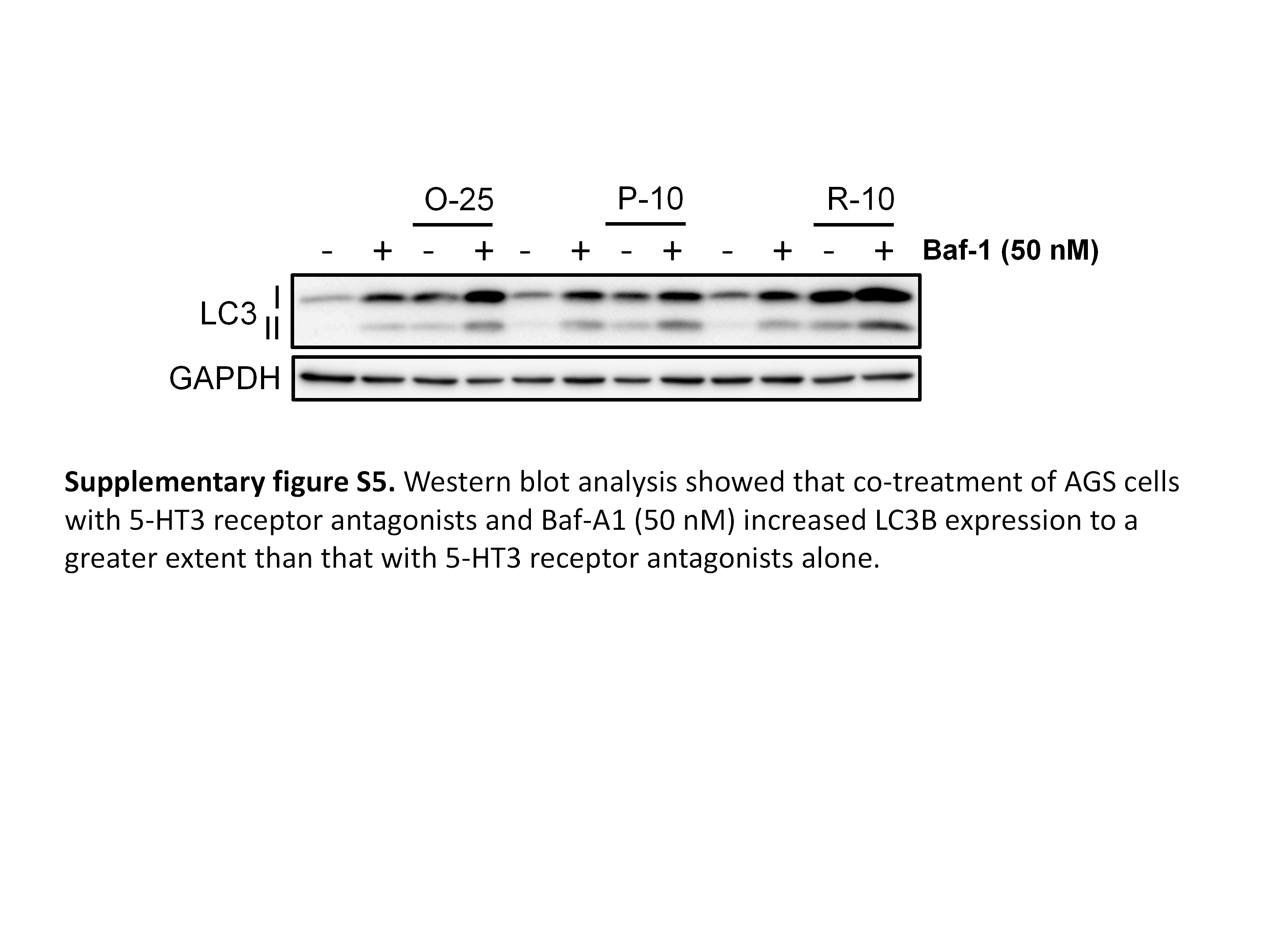

Supplement: Supplementary file 1 [file ijms-26-10039-s001.zip › Supplementary figures_05.png]

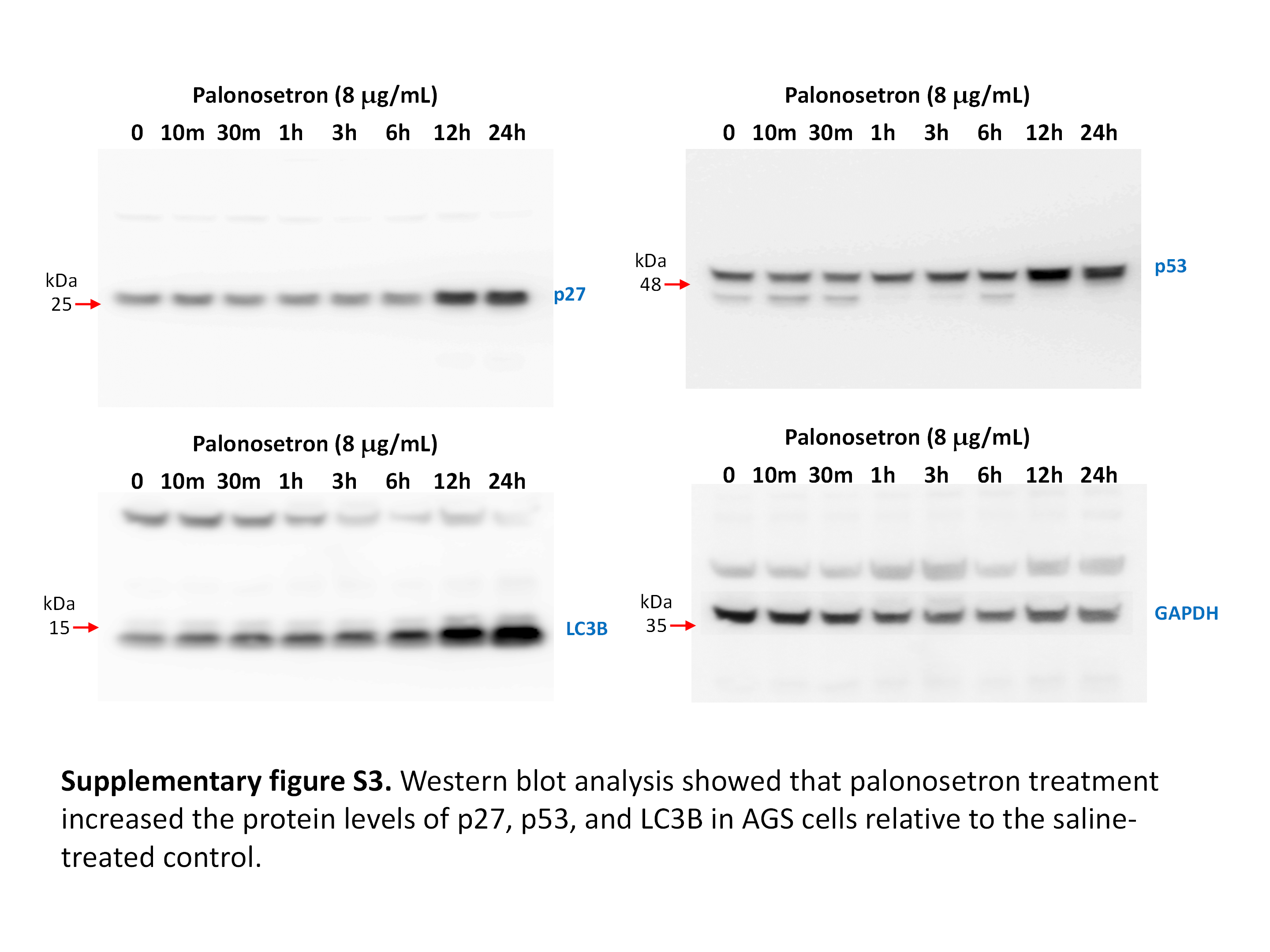

Supplement: Supplementary file 1 [file ijms-26-10039-s001.zip › Supplementary figures_03.png]

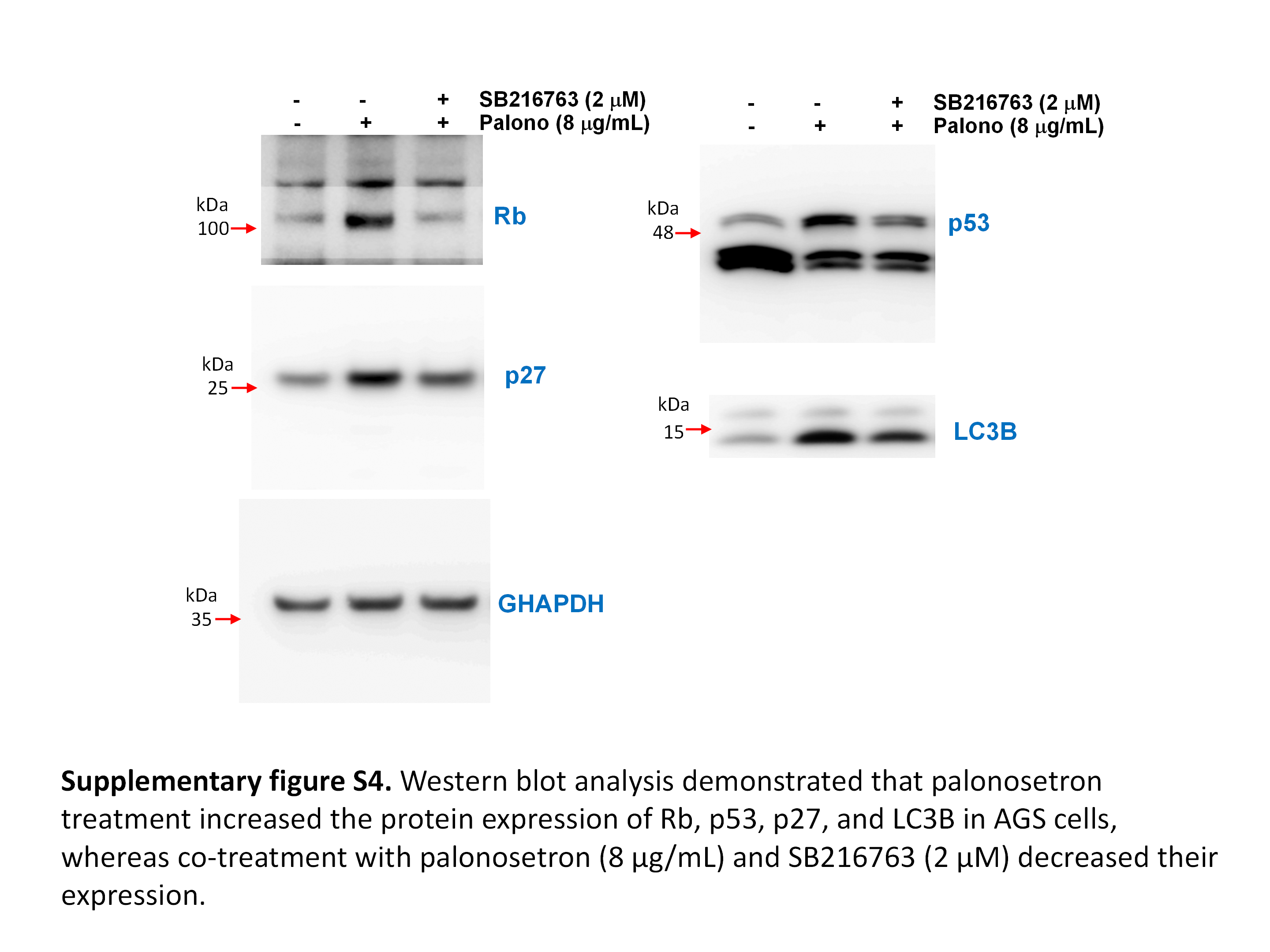

Supplement: Supplementary file 1 [file ijms-26-10039-s001.zip › Supplementary figures_04.png]

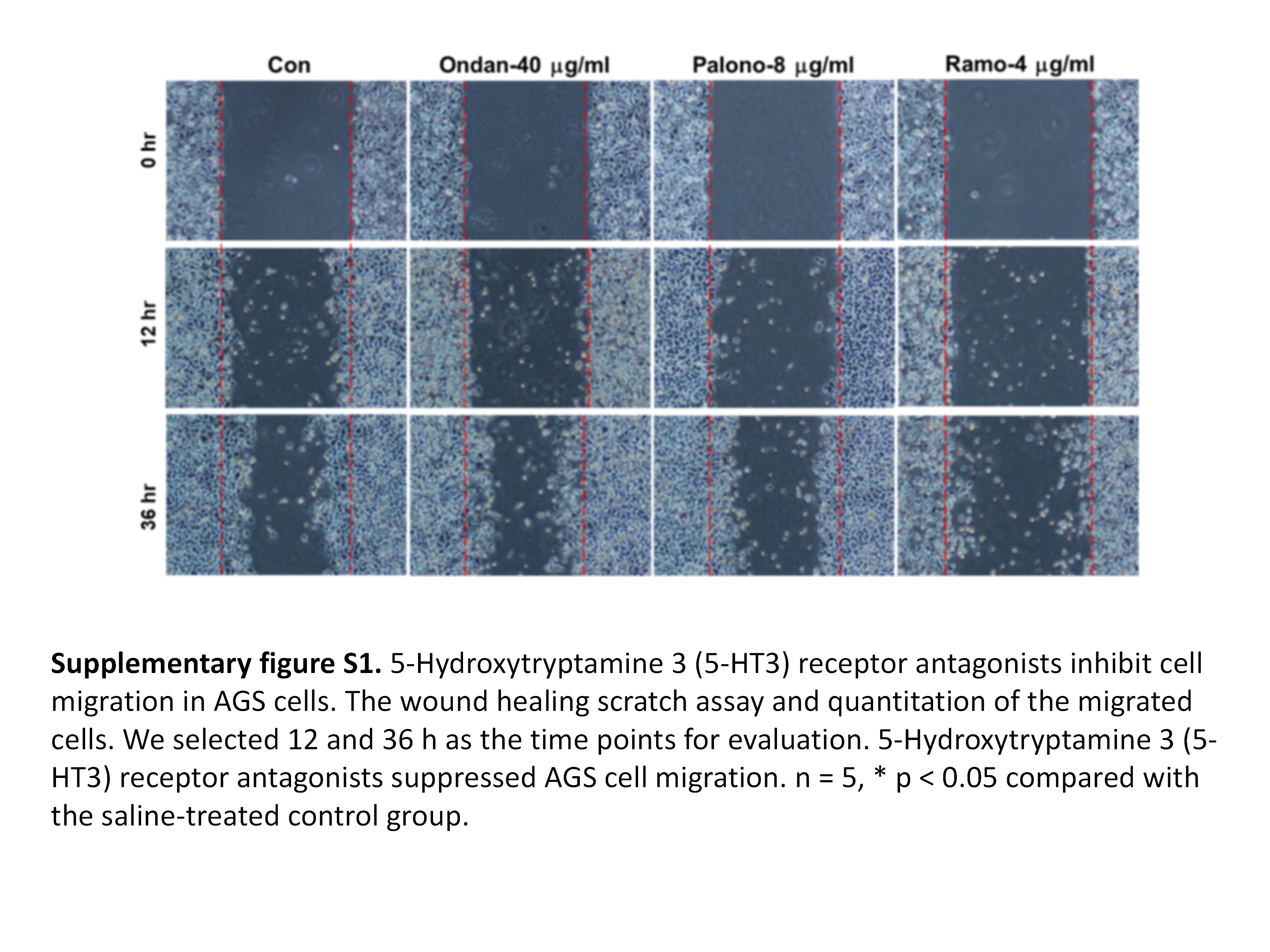

Supplement: Supplementary file 1 [file ijms-26-10039-s001.zip › Supplementary figures_01.png]

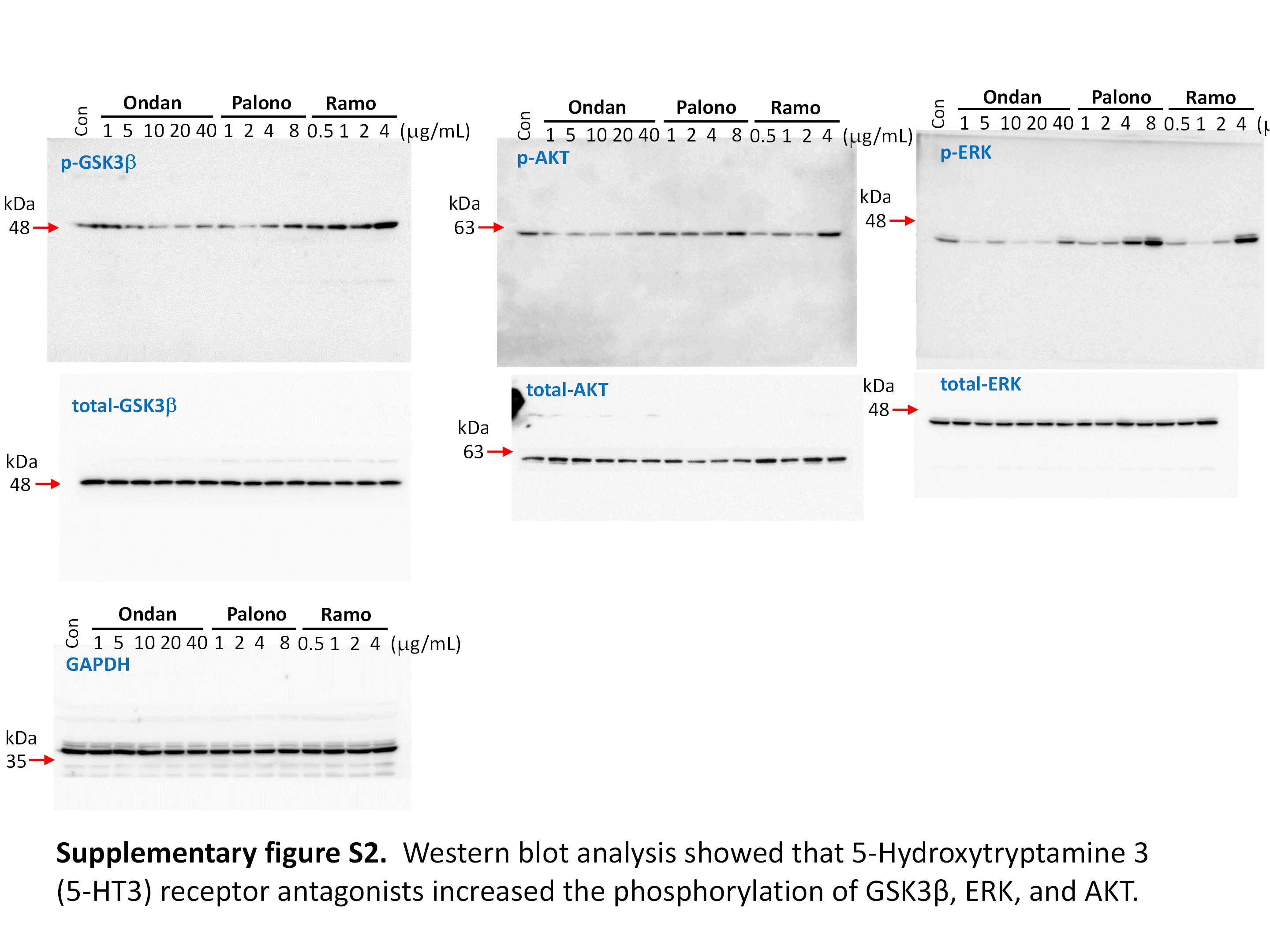

Supplement: Supplementary file 1 [file ijms-26-10039-s001.zip › Supplementary figures_02.png]
